# The Use of Hybrid Lumbar Puncture Simulation to Teach Entrustable Professional Activities During a Medical Student Neurology Clerkship

**DOI:** 10.15694/mep.2020.000266.2

**Published:** 2021-09-03

**Authors:** Claire Yanta, Laurie Knepper, Reed Van Deusen, Kristine Ruppert

**Affiliations:** 1 University of Pittsburgh School of Medicine; 2 University of Pittsburgh Clinical and Translational Science Institute

**Keywords:** hybrid simulation, lumbar puncture, Entrustable Professional Activity

## Abstract

This article was migrated. The article was marked as recommended.

**Background:** In 2014, the Association of American Medical Colleges (AAMC) published a list of 13 Entrustable Professional Activities (EPAs) that medical school graduates should be able to perform upon starting residency. The University of Pittsburgh School of Medicine (UPSOM) has surveyed our neurology clerkship students in regard to EPAs since 2017; according to this data we have been deficient in addressing EPAs 4 (enter and discuss orders/prescriptions), 11 (obtain informed consent for tests and/or procedures), and 12 (perform general procedures of a physician). We therefore developed a hybrid simulation experience encompassing these three skills, centered around lumbar puncture (LP).

**Methods:** We created a hybrid LP simulation for students on the neurology clerkship encompassing EPAs 4, 11, and 12. Students first obtained informed consent for LP from a Standardized Patient, then performed LP on a specialized manikin. They then entered orders on CSF into a simulated patient chart. Real-time feedback was provided for all three components. Students filled out surveys to assess their perceived confidence and skill with these activities both pre- and post-simulation.

**Results:** The percentage of students who increased their confidence with LP from minimal or less to average or more was 58.24%, 38.47%, and 26.38% for LP, informed consent, and order entry, respectively. The percentage of students who improved from not being able to perform/needing significant supervision to being able to perform with minimal supervision/ independently was 25.27%, 47.25% and 28.58%, for LP, informed consent, and order entry, respectively. These differences were all statistically significant (p

**Conclusions/Significance:** Hybrid LP simulation was effective in increasing medical student confidence and perceived skill with EPAs 4, 11, and 12.

## Introduction

According to a 2016 survey, 57% of US medical school graduates do not perform a lumbar puncture-a crucial diagnostic procedure for neurological conditions- prior to graduation ( Barr and Graffeo, 2016). Likewise, a 2019 survey of German medical students found that, out of 14 common medical procedures, the students endorsed the least confidence with performing a lumbar puncture ( von Cranach, Backhaus and Brich, 2019). The Association of American Medical Colleges (AAMC) recognizes the importance of procedural skill in medical education, as reflected in its list of 13 Entrustable Professional Activities (EPAs) that students should be able to perform upon starting residency ( AAMC, 2014).

In the 4-year United States (US) medical education system, schools require their students to complete clinical experiences involving direct patient care-known as clerkships-typically beginning in the students’ third year. At the University of Pittsburgh School of Medicine (UPSOM), we introduced the EPAs to our students on our required neurology clerkship in 2015 and have given the students yearly surveys to determine how well we teach these skills during the clerkship. Based on this survey data, we had previously developed a curriculum centered around note-writing to teach EPAs 1 (“Gather a history and perform a physical examination”), 2 (“Prioritize a differential diagnosis following a clinical encounter”), and 5 (“Document a clinical encounter in the patient record”). Since implementing this curriculum, the percentage of students who state that our clerkship teaches these activities has been 99-100% in all three activities. Our findings have been presented in poster form at the 2018 World Summit on Competency-Based Medical Education in Basel, Switzerland ( Knepper *et al.* 2018).

According to our students, our neurology clerkship has remained deficient in addressing EPAs 4, 11, and 12, which are defined as follows:

• EPA 4: Enter and discuss orders/prescriptions
• EPA 11: Obtain informed consent for tests and/or procedures
• EPA 12: Perform general procedures of a physician

According to our survey data, 75% of our students reported having entered and discussed orders, 53% of our students reported participating in obtaining informed consent, and 78% reported performing “general procedures of a physician”. The number for LP, specifically, was not recorded.

There are many reasons for these low performance rates. For one, Pennsylvania state law mandates that informed consent must be obtained by a physician, thus students are not afforded real-life experience in obtaining informed consent on their own. In addition, there are multiple possible sites at which our students may have their clinical experiences; while we strive for site equivalence, there are certain services where lumbar puncture procedures are more commonly done than others. Finally, different services also have different degrees of involvement in order entry (primary neurology service vs consultation services, for example). These problems are not unique to our own institution but are experienced, to varying degrees, by neurology clerkship directors across the country ( Safdieh *et al*., 2018).

Simulation-based training has been recommended by the American Academy of Neurology as a potential way to alleviate novice stress levels before and during lumbar puncture procedures ( Henriksen *et al.*, 2018). 53% of neurology clerkships have LP simulation exercises during their neurology clerkships ( Safdieh *et al*., 2018). To date, there are few studies that looked at whether these simulations improved student confidence with performing LP. A 2017 study of 24 second- and third-year medical students who received simulation-based training on multiple procedural skills including lumbar puncture showed that student self-reported confidence and procedural performance scores significantly improved following their training sessions ( Toy *et al*., 2017). Similarly, a 2018 study of 20 senior medical students who received small group simulation-based training in multiple clinical skills including lumbar puncture showed significant improvement in their skill performance as compared to PGY-1 (postgraduate year 1; i.e. first year following medical school graduation) historical controls ( Wayne *et al*., 2018).

The results of these studies have been positive, though it is important to note that the outcomes reported here were based on skills assessments and survey data immediately following the simulation experiences. There is no data available as to whether these simulations advance skill and improve self-efficacy in real-world settings. Furthermore, to our knowledge, there are no other high-fidelity, comprehensive simulation experiences that also incorporate obtaining informed consent from standardized patients and entering orders into the electronic medical record (EMR). Hybrid simulation has, however, demonstrated to be both feasible and effective for medical student training of gynecologic procedures such as intrauterine device insertion and Papanicolaou tests as well as breast examinations ( Amerjee *et al*., 2018; Seo *et al*., 2018; Nassif *et al*., 2019). We therefore developed a hybrid simulation experience encompassing all three of these crucial skills and centered around LP. It was our belief that this innovative hybrid simulation model would translate into improved student confidence and capability in a similar real-life scenario. Preliminary data was accepted for presentation in poster form at the Association for Medical Education in Europe 2020: The Virtual Experience ( Yanta *et al.*, 2020). We now present our final data.

## Methods

This project was approved by the UPSOM Research on Medical Students Committee and University of Pittsburgh Institutional Review Board. At the beginning of the 2019-2020 academic year, we first distributed a survey to the rising MS-4 (fourth-year medical student) class to determine how often they participated in informed consent, electronic order entry, and lumbar puncture during their third-year clerkships (Supplementary File 1). We then incorporated our simulation into the clerkship curriculum. During the clerkship orientation, the students received a tutorial on obtaining informed consent and entering orders into the EMR from a faculty preceptor. Within the first four days of the clerkship, the students completed an online Likert-style survey assessing their level of confidence and perceived skill with these activities (Supplementary File 2). The design of this survey was based on previously published instruments ( Dehmer *et al*., 2013; von Cranach, Backhaus and Brich, 2019).

On the fifth day of the 28-day clerkship, the students then gathered for the hybrid simulation experience. During this experience, they first simulated obtaining informed consent for a lumbar puncture from a standardized patient (SP) with a chief complaint of monocular vision loss concerning for optic neuritis. Feedback was immediately provided to the students based on an informed consent checklist provided to the SPs (Supplementary File 3). The students then received instruction from neurology faculty or senior resident preceptors on performing LP with the use of a specialized manikin. They were then able to practice the procedure with real-time feedback provided by preceptors. The students then entered orders on the “CSF” into a simulated EMR, again obtaining real-time feedback from preceptors.

Following the simulation exercise, the students filled out a similar Likert-style survey to assess their confidence and perceived skill with these three activities (Supplementary File 4). At the end of the four-week clerkship, the students filled out an additional survey to determine how many of them had live-patient experiences with these skills (Supplementary File 5). Those students who performed these skills on live patients identified the clinical preceptors who supervised them. Those supervisors, in turn, were asked to submit an evaluation of the students’ roles in those crucial skills (Supplementary File 6). All this data was then de-identified and analyzed in aggregate, stratified by time of academic year (block 1-5 vs block 6 onward). P values were calculated using Fisher’s exact test. At the end of the academic year, this same cohort of students was then asked to complete an identical online survey to that of the previous rising senior class (Supplementary File 1). These results were compared to the survey results of the prior rising senior class.

## Results

Of the 128 students who completed the neurology clerkship during the 2019-2020 academic year, 84 completed the pre-simulation survey, 70 completed the post-simulation survey and 49 completed the end of clerkship survey.

### Confidence and Perceived Skill with Lumbar Puncture

In the pre-simulation survey, the percentage of students who endorsed average or better confidence with LP was 5.95%. Following the simulation, this percentage improved to 90%. The proportion of students who endorsed average confidence increased by a net 55.48 percentage points, above average confidence increased by 27.14 percentage points, and maximal confidence increased by 1.43 percentage points ( Table 1). When broken down by respondent, the percentage of students whose confidence with LP improved from nil/minimal to average or better was 58.24%. This increase was statistically significant (p < 0.0001).

In addition to self-confidence, we also asked the students to rate their perceived skill with LP both before and following the simulation. In the pre-simulation survey, the percentage of students who reported that they were able to perform LP with minimal or no assistance was 0%. Following the simulation, this percentage improved to 38.57%. The proportion of students who reported requiring minimal supervision for LP increased by a net 38.57 percentage points ( Table 2). When we looked at individual respondents, the percentage of students whose perceived skill with LP improved from not being able to perform/needing significant supervision to being able to perform with minimal supervision or independently was 25.27%. This increase was statistically significant (p < 0.0001).

**Table 1:** Student Confidence Pre- vs Post-Simulation (%)

	LP: Pre	LP: Post	Net ∆	Informed Consent: Pre	Informed Consent: Post	Net ∆	Electronic Order Entry: Pre	Electronic Order Entry: Post	Net ∆
**No confidence**	63.10	0.00	-63.10	11.90	1.43	- 10.47	13.1	0.00	-13.10
**Minimal Confidence**	30.95	10.00	-20.95	41.67	1.43	-40.24	30.95	10.00	-20.95
**Average Confidence**	5.95	61.43	+55.48	32.14	30.00	-2.14	30.95	42.86	+11.91
**Above Average Confidence**	0.00	27.14	+27.14	13.10	60.00	+46.90	21.43	41.43	+20.00
**Maximal Confidence**	0.00	1.43	+1.43	1.19	7.14	+5.95	3.57	5.71	+2.14

**Table 2:** Student Perceived Skill Pre- vs Post-Simulation (%)

	LP: Pre	LP: Post	Net ∆	Informed Consent: Pre	Informed Consent: Post	Net ∆	Electronic Order Entry: Pre	Electronic Order Entry: Post	Net ∆
**Unable to Perform**	45.74	0.00	-45.74	5.95	0.00	-5.95	4.76	0.00	-4.76
**Requires Significant Supervision**	54.76	61.43	+6.67	63.10	4.29	-58.81	44.05	10.00	-34.05
**Requires Minimal Supervision**	0.00	38.57	+38.57	28.57	70.00	+41.43	41.67	71.43	+29.76
**Can Perform Independently**	0.00	0.00	0.00	2.38	25.71	+23.33	9.52	18.57	+9.05


### Confidence and Perceived Skill with Obtaining Informed Consent

In the pre-simulation survey, the percentage of students who endorsed average or better confidence with obtaining informed consent was 46.43%. Following the simulation, this percentage improved to 97.14%. Above average confidence with obtaining informed consent increased by 46.90 percentage points, and maximal confidence increased by 5.95 percentage points ( Table 1). When broken down by respondent, the percentage of students whose confidence with obtaining informed consent improved from nil/minimal to average or better was 38.47%. This increase was statistically significant (p < 0.0001).

In the pre-simulation survey, the percentage of students who reported that they were able to obtain informed consent with minimal or no assistance was 30.95%. Following the simulation, this percentage improved to 95.71%. The proportion of students who reported requiring minimal supervision with obtaining informed consent increased by 41.43 percentage points, and the proportion of students who felt able to perform this skill independently increased by 23.33 percentage points ( Table 2). When we looked at individual respondents, the percentage of students whose perceived skill with obtaining informed consent improved from not being able to perform/needing significant supervision to being able to perform with minimal supervision or independently was 47.25%. This increase was statistically significant (p < 0.0001).

### Confidence and Perceived Skill with Electronic Order Entry

In the pre-simulation survey, the percentage of students who endorsed average or better confidence with electronic order entry was 55.95%. Following the simulation, this percentage improved to 90%. The proportion of students who reported average confidence with electronic order entry increased by 11.91 percentage points, above average confidence increased by 20.00 percentage points, and maximal confidence increased by 2.14 percentage points ( Table 1). When broken down by respondent, the percentage of students whose confidence with electronic order entry improved from nil/minimal to average or better was 26.38%. This increase was statistically significant (p < 0.0001).

In the pre-simulation survey, the percentage of students who reported that they were able to perform electronic order entry with minimal or no assistance was 51.19%. Following the simulation, this percentage improved to 90%. The proportion of students who felt that they required minimal supervision for electronic order entry increased by 29.76 percentage points, and the proportion of students who felt able to perform the skill independently increased by 9.05 percentage points ( Table 2). When we looked at individual respondents, the percentage of students whose perceived skill with electronic order entry improved from not being able to perform/needing significant supervision to being able to perform with minimal supervision or independently was 28.58%. This increase was statistically significant (p < 0.0001).

### Effect of Clerkship Timing within Academic Year on Results

To determine whether the students’ cumulative clinical experience in other clerkships affected the utility of the simulation, we stratified the survey results based on when in the academic year they rotated through the neurology clerkship (first half of the academic year vs second half). Between these groups, there was no statistically significant difference in the degree of improvement following the simulation regarding confidence and perceived skill with LP or informed consent (p values 0.13 - 1). There was, however, a significant difference between these two groups regarding confidence and skill with electronic order entry. The percentage of students whose confidence in electronic order entry improved from nil/minimal to average or more was 48.15% in the early group and 19.29% in the late group (p = 0.0266). The percentage of students whose perceived skill with electronic order entry improved from not being able to perform/needing significant supervision to being able to perform with minimal supervision or independently was 55.56% in the early group and 19.29% in the late group (p = 0.0052).

### Preceptor Evaluation of Student Skills with Live Patients

Out of 49 respondents to the end-of-clerkship survey, 27 (55%) reported participating in at least one LP, and 2 (4%) reported participating in 3 or more. 18 (37%) respondents reported participating in at least one informed consent discussion, and 2 (4%) reported participating in 3 or more. 15 (31%) respondents reported participating in at least one electronic order entry, and 6 (12%) reported participating in 3 or more. Regarding LP performance, seven clinical preceptors responded to the student performance evaluation request, with two preceptors declining to score their students on the basis that the students only observed the procedure and did not actively participate. Of the five remaining evaluations, three of them gave their students a score of 2 (able to perform with significant supervision), and two of them gave their students a score of 3 (able to perform with minimal supervision). Regarding informed consent performance, six clinical preceptors responded to the student performance evaluation request; three of these preceptors declined to score their students. Of the remaining three, two of them gave their students a score of 4 (able to perform independently). The other preceptor, while not providing a numerical score, wrote that “[the student] was thorough and completed the consent verbiage without assistance”. Regarding order entry, six clinical preceptors responded to the student performance evaluation request; three declined to score their students. Of the remaining three preceptors, one gave their student a score of 2, one gave their student a score of 3, and one gave their student a score of 4.

### Comparison of Real-World Experience of Simulation Cohort vs. No-Simulation Cohort

To determine whether or not the simulation experience enabled the students to better participate in these skills in real-world settings, we compared the results of the survey sent to the MS-4s at the beginning of the 2019-2020 academic year (i.e. students who did not participate in the simulation) to the results of the same survey sent to rising MS-4s at the beginning of the 2020-2021 academic year. 25 students completed the 2019-2020 survey, and 17 students completed the 2020-2021 survey. One respondent to the 2019-2020 survey reported not yet completing the neurology clerkship. Of the 25 students who completed the 2019-2020 survey, 13 (52%) reported participating in at least one LP, 20 students (80%) reported participating in at least one informed consent discussion, and 21 students (84%) reported participating in at least one electronic order entry. By comparison, of the 17 students who completed the 2020-2021 survey, 11 (65%) reported participating in at least one LP, 16 students (94%) reported participating in at least one informed consent discussion, and 17 students (100%) reported participating in at least one electronic order entry (Tables 3 and 4).

**Table 3:** MS-4 Survey Responses 2019-2020 (n=25) (%)

	LP	Informed Consent	Electronic Order Entry
**0**	12 (48.00)	5 (20.00)	4 (16.00)
**1-2**	13 (52.00)	11 (44.00)	4 (16.00)
**3+**	0 (0.00)	9 (36.00)	17 68.00)

**Table 4:** MS-4 Survey Responses 2020-2021 (n=17) (%)

	LP	Informed Consent	Electronic Order Entry
**0**	6 (35.29)	1 (5.88)	0 (0.00)
**1-2**	10 (58.82)	10 (58.82)	6 (35.29)
**3+**	1 (5.88)	6 (35.29)	11 (64.71)

## Discussion

Our data demonstrate that our hybrid lumbar puncture simulation was effective at improving students’ confidence and perceived skill with obtaining informed consent, performing LP, and entering orders into the EMR. These effects were seen independent of the clinical experience of the students as measured by the time of academic year they had their clerkship, particularly regarding obtaining informed consent and in performing LP. There was a statistically significant attrition in the improvement in confidence and perceived skill with electronic order entry in the late group as opposed to the early group. This finding is likely explained by the fact that rising fourth-year students reported more experience with order entry during their third-year clinical curriculum than they did with LP or with informed consent discussions. Correspondingly, students who rotated through the neurology clerkship later in the year had higher pre-simulation confidence and perceived skill scores than their earlier counterparts. Still, even in later groups, 19.29% of students had significant improvement in their confidence and perceived skill following our simulation.

Our data regarding improved real-world improvement following the simulation was hampered by low survey response rate. Only 49 students completed the end-of-clerkship survey, as opposed to the 84 who filled out the pre-simulation survey and 70 who filled out the post-simulation survey. Likewise, very few clinical preceptors responded to the student performance evaluation request (7 responses out of 27 student reports for LP, 6 responses out of 19 student reports for informed consent, and 6 responses out of 16 student reports for order entry), and relatively few students completed the class-wide surveys sent at the beginning of academic years 2019-2020 and 2020-2021. When taken descriptively, however, it can at least be said that the students were observed as being able to complete these skills with varying degrees of supervision, and rising MS-4s who participated in the simulation reported more real-world experience with these skills than their counterparts who did not participate in the simulation.

In conclusion, we have demonstrated that hybrid LP simulation is a feasible and effective way to teach lumbar puncture, informed consent, and electronic order entry. This simulation improves confidence and perceived skill with all three of these activities regardless of the clinical experience of the student. Furthermore, there is some suggestion from our data that this simulation also promotes competency with these skills in real-world settings.

## Take Home Messages

• Lumbar puncture is currently underperformed by medical students prior to graduation.
• Lumbar puncture, informed consent discussions, and electronic order entry are three skills that medical school graduates should be able to perform upon entry into residency.
• Hybrid lumbar puncture simulation is feasible and effective in teaching medical students how to perform lumbar puncture, obtain informed consent, and enter orders into the electronic medical record.

## Notes On Contributors

**Claire Yanta**, MD: Dr. Yanta is an Assistant Professor of Neurology at the University of Pittsburgh School of Medicine in Pittsburgh, PA, USA. She is the Director of the UPSOM MS-1 Neuroscience Course and is the Assistant Director of the UPSOM Neurology Clerkship.

**Laurie Knepper**, MD: Dr. Knepper is an Associate Professor of Neurology at the University of Pittsburgh School of Medicine in Pittsburgh, PA, USA. She is the Director of the UPSOM Neurology Clerkship and is the Vice Chair of Education in the UPMC Department of Neurology.

**Reed Van Deusen**, MD: Dr. Van Deusen is an Associate Professor of Medicine at the University of Pittsburgh School of Medicine in Pittsburgh, PA, USA. He is the Assistant Dean for Human-Based Simulation Education and the Medical Director of the Standardized Patient Program at UPSOM.

**Kristine Ruppert**, DrPH: Dr. Ruppert is an Assistant Professor of Epidemiology at the University of Pittsburgh Graduate School of Public Health. She also works with the University of Pittsburgh Clinical and Translational Science Institute to facilitate research within the health sciences at the University of Pittsburgh.
