# Supplementary material for: The Use of Hybrid Lumbar Puncture Simulation to Teach Entrustable Professional Activities During a Medical Student Neurology Clerkship
Source: MedEdPublish (2016). 2021 Sep 3;9:266. Originally published 2020 Nov 27. [Version 2] doi: 10.15694/mep.2020.000266.2 (PMC10697465; doi:10.15694/mep.2020.000266.2)
Supplement: Peer review history

# Reviewer report: Reviewer response for version 2

**DOI:** 10.21956/mep.20286.r31500

**Published:** 2021-10-07

**Authors:** Dimitri Parra

**Affiliations:** 1 The Hospital for Sick Children

This review has been migrated. The reviewer awarded 4 stars out of 5 The authors present their experience with hybrid lumbar puncture simulation to teach EPA to medical students. Very interesting article. The review has significantly improved the paper.This work contributes to the field by showing creative alternatives to work with deficiencies in EPAs at different stages in medical education. I believe that this will also be useful in post graduate education. I think the study design is adequate and I hope in future publications the portion based on the real-life experiences and assessment has more evidence. I think that is the key as, even though is valid, the student’s self-assessment has limitations, and demonstrating quality improvement and decrease of health-related costs is the way to go to demonstrate the health care administrators that it is needed to include more simulated experiences to improve patients’ outcomes and organizational efficiency.Congrats for the paper and I hope that the authors continue doing research in similar areas.

Reviewer Expertise:

NA

No decision status is available

# Reviewer report: Reviewer response for version 2

**DOI:** 10.21956/mep.20286.r31498

**Published:** 2021-09-14

**Authors:** Ken Masters

**Affiliations:** 1 Sultan Qaboos University

This review has been migrated. The reviewer awarded 5 stars out of 5 The authors have responded well to my comments on Version 1 of the paper (most of my comments dealt with layout and flow). The paper is now much easier to read and follow, and is a useful contribution to the field. Thank you.

Reviewer Expertise:

NA

No decision status is available

# Reviewer report: Reviewer response for version 2

**DOI:** 10.21956/mep.20286.r31499

**Published:** 2021-09-07

**Authors:** BALAJI ARUMUGAM

**Affiliations:** 1 TAGORE MEDICAL COLLEGE AND HOSPITAL

This review has been migrated. The reviewer awarded 4 stars out of 5 Simulation based medical education is defined as any educational activity that utilizes simulation aides to replicate clinical scenarios. Simulation tools serve as an alternative to real patients. A trainee can make mistakes and learn from them without the fear of harming the patient. But Hybrid simulation defined as the use of two or more simulation modalities within the same simulation session. An Entrustable Professional Activity is a key task of a discipline (i.e. specialty or subspecialty) that an individual can be trusted to perform in a given health care context, once sufficient competence has been demonstrated.The study was conducted and well handled using an educational tool (hybrid Simulation) to assess the learning among the fourth year medical students. The EPAs of 4, 11 and 12 but the sequence of EPAs assessed in this simulation is obtaining the informed consent (11), performing a procedure (12) and entering the orders (4). This Hybrid Simulation included three EPA competencies included as a single simulation scenarios for which the before and after surveys of the medical students on perceptions of confidence and performance of skill was obtained. Of the 128 students who completed the neurology clerkship during the 2019-2020 academic year, 84 completed the pre-simulation survey, 70 completed the post-simulation survey and 49 completed the end of clerkship survey. The attrition seems to be high. Question arises as the reviewer in this portion? At the end of the academic year, this same cohort of students was then asked to complete an identical online survey to that of the previous rising senior class (Supplementary File 1). These results were compared to the survey results of the prior rising senior class.This result is not within the objective of this study.

Reviewer Expertise:

NA

No decision status is available

# Reviewer report: Reviewer response for version 1

**DOI:** 10.21956/mep.19144.r27677

**Published:** 2021-07-13

**Authors:** Sateesh Babu Arja

**Affiliations:** 1 Avalon University School of Medicine

This review has been migrated. The reviewer awarded 4 stars out of 5 Thank you for submitting this manuscript for MedEdPublish. I enjoyed reading this paper. It is good to see this paper in the current context of Competency-based Medical Education (CBME) and Entrustable Professional Activities (EPAs). The authors presented the problem statement and the literature gap. The research methodology and data analysis are appropriate. The conclusions drawn were coherent with the results presented. But it could have been better, especially with long paragraphs, and readers might require to go back and forth. I am also skeptical of the transferability of knowledge from the simulation world to the real world, which could be an issue for any kind of simulation. And also, there is a drop in survey response rate at the end of the clerkship, which the authors also mentioned.

Reviewer Expertise:

NA

No decision status is available

# Reviewer report: Reviewer response for version 1

**DOI:** 10.21956/mep.19144.r27676

**Published:** 2021-02-12

**Authors:** Ken Masters

**Affiliations:** 1 Sultan Qaboos University

This review has been migrated. The reviewer awarded 3 stars out of 5 An interesting paper on the use of hybrid lumbar puncture simulation to teach EPAs during a medical student neurology clerkship. The authors begin by clearly identifying a problem that they need to solve, namely that three EPAs are not being adequately addressed in their current training. Although there are good reasons for this, some of which are beyond their control, it remains a problem to be solved. They then go on to describe a simulation training that has led to positive results.The main issue with the paper, however, is in the presentation, and I elaborate on this below:• Some of the paragraphs are really long, and the reader is met with a wall of text. It would be preferable if these longer paragraphs could be broken into smaller paragraphs. This is important in the opening paragraphs, but also the paragraphs in the Results, where the reader is met with ideas and numbers that seem to flow into each other. These paragraphs can also benefit from sub-headings, to guide the reader through the text. The authors should bear in mind that they have conducted a study with multiple dimensions and results, and the aim is to ensure that the readers understand exactly what happened, and what the results were. Otherwise, the numbers get lost in a blur.• It also does not help that the text in the Results refers to the EPAs by their names, but the headings in Table 1 and 2 refers to them by their number (even though there is a key at the bottom). It would be useful to be consistent in the convention, so I recommend that the table headings (can be split across two columns) give the name so that readers can more easily match what is being said in the text to what is being displayed in the table.• Similarly, it would help the reader if the authors reported the results in the same order. In the text, they are displayed in LP, informed consent, order entry. In Table 1 and 2, they run order entry, informed consent and LP. Table 3 and 4 uses names, and runs: LP, informed consent, order entry, but also introduces EMR into the heading. A reader would now ask themselves if these are somehow different from the other order entries. Or might just stop reading.• Other issues (again, of clarity) have been raised by the previous reviewer, so there is no need to repeat them here.So, the study appears to be worth-while and has been well-conducted. The presentation of the paper, however, makes it difficult to read, and I can imagine most readers’ eyes glazing over and not completing the paper. I would like to see Version 2 of the paper, in which the authors address the issue of presentation, perhaps asking colleagues who are not familiar with the project to pre-screen it.

Reviewer Expertise:

NA

No decision status is available

# Reviewer report: Reviewer response for version 1

**DOI:** 10.21956/mep.19144.r27678

**Published:** 2020-12-01

**Authors:** Mary Higgins

**Affiliations:** 1 University College Dublin, National Maternity Hospital

This review has been migrated. The reviewer awarded 3 stars out of 5 Thank you for this interesting study. I am fascinated by the preceptor who signed off a students as being able to perform this independently. While you report that 54% of students had done at least one LP, what was the range? Can you also clarify the following for an international audience: PGY-1 (postgraduate year one? first year post graduation?)Clerkships (clinical year student?)MS-4 (fourth year student?)In the introduction what are EPAs 1,2,5? How many EPAs are there?

Reviewer Expertise:

NA

No decision status is available
